# A Decoy-Receptor Approach Using Nicotinic Acetylcholine Receptor Mimics Reveals Their Potential as Novel Therapeutics Against Neurotoxic Snakebite

**DOI:** 10.3389/fphar.2019.00848

**Published:** 2019-07-30

**Authors:** Laura-Oana Albulescu, Taline Kazandjian, Julien Slagboom, Ben Bruyneel, Stuart Ainsworth, Jaffer Alsolaiss, Simon C. Wagstaff, Gareth Whiteley, Robert A. Harrison, Chris Ulens, Jeroen Kool, Nicholas R. Casewell

**Affiliations:** ^1^Centre for Snakebite Research & Interventions, Liverpool School of Tropical Medicine, Liverpool, United Kingdom; ^2^AIMMS Division of BioMolecular Analysis, Vrije Universiteit Amsterdam, Amsterdam, Netherlands; ^3^Bioinformatics Unit, Liverpool School of Tropical Medicine, Liverpool, United Kingdom; ^4^Centre for Drugs and Diagnostics, Liverpool School of Tropical Medicine, Liverpool, United Kingdom; ^5^Laboratory of Structural Neurobiology, Department of Cellular and Molecular Medicine, Faculty of Medicine, KU Leuven, Leuven, Belgium

**Keywords:** nicotinic acetylcholine receptors (nAChR), long-chain three-finger toxins (3FTx), acetylcholine binding proteins (AChBPs), snake venom neurotoxins, envenoming, therapeutics

## Abstract

Snakebite is a neglected tropical disease that causes 138,000 deaths each year. Neurotoxic snake venoms contain small neurotoxins, including three-finger toxins (3FTxs), which can cause rapid paralysis in snakebite victims by blocking postsynaptic transmission *via* nicotinic acetylcholine receptors (nAChRs). These toxins are typically weakly immunogenic and thus are often not effectively targeted by current polyclonal antivenom therapies. We investigated whether nAChR mimics, also known as acetylcholine binding proteins (AChBPs), could effectively capture 3FTxs and therefore be developed as a novel class of snake-generic therapeutics for combatting neurotoxic envenoming. First, we identified the binding specificities of 3FTx from various medically important elapid snake venoms to nAChR using two recombinant nAChR mimics: the AChBP from *Lymnaea stagnalis* and a humanized neuronal α7 version (α7-AChBP). We next characterized these AChBP-bound and unbound fractions using SDS-PAGE and mass spectrometry. Interestingly, both mimics effectively captured long-chain 3FTxs from multiple snake species but largely failed to capture the highly related short-chain 3FTxs, suggesting a high level of binding specificity. We next investigated whether nAChR mimics could be used as snakebite therapeutics. We showed that while α7-AChBP alone did not protect against *Naja haje* (Egyptian cobra) venom lethality *in vivo*, it significantly prolonged survival times when coadministered with a nonprotective dose of antivenom. Thus, nAChR mimics are capable of neutralizing specific venom toxins and may be useful adjunct therapeutics for improving the safety and affordability of existing snakebite treatments by reducing therapeutic doses. Our findings justify exploring the future development of AChBPs as potential snakebite treatments.

## Introduction

Snakebite is a neglected tropical disease that results in high mortality (138,000 deaths per annum) and morbidity (∼400,000 cases per annum), and it is the rural impoverished people of the tropics who suffer the greatest burden (Gutiérrez et al., 2017). Elapids (family Elapidae) are one of the main groups of medically important snakes responsible for severe envenoming and include the cobras (*Naja* spp.), kraits (*Bungarus* spp.), mambas (*Dendroaspis* spp.), and coral snakes (*Micrurus* spp.). Elapid envenoming often causes postsynaptic neurotoxic effects by blocking neuromuscular transmission that can culminate in respiratory paralysis and death (Gutiérrez et al., 2017). Three-finger toxins (3FTxs) play a key role, with many competitively binding to postsynaptic neuromuscular and neuronal nicotinic acetylcholine receptors (nAChRs) to inhibit the binding of acetylcholine (Chang, 1979). Importantly, ever since the discovery of α-bungarotoxin, snake venom neurotoxins have been extensively employed for characterizing nAChRs and understanding the basis of neurotransmission. Therefore, a comprehensive knowledge of their targets and mechanisms of action remains important for a range of biological disciplines.

Three-finger toxins are one of many snake venom toxin types that are encoded by a multilocus gene family (Casewell et al., 2013). The frequent duplication of toxin-encoding genes coupled with bursts of accelerated evolution results in snake venom composition varying at every taxonomic level, including inter- and intraspecifically (Chippaux et al., 1991; Casewell et al., 2013; Casewell et al., 2014). Moreover, elapid venom proteomes are usually dominated by 3FTxs, which typically consist of numerous isoforms that comprise over 60% of venom proteins (Tasoulis and Isbister, 2017). These are small disulfide-bond rich proteins with a globular core and three β-stranded loops that extend from the core as “fingers” and can be classified into short-chain, long-chain, weak and nonconventional neurotoxins, cardiotoxins, and others (Kessler et al., 2017). Short-chain 3FTxs are small proteins consisting of 60–62 amino acid residues that contain four disulfide bridges, while long-chain α-neurotoxins (long-chain 3FTxs) have 66–74 amino acid residues and display an additional disulfide bond.

The small size (∼7–12 kDa) of these potent neurotoxins poses a therapeutic challenge—they are weakly immunogenic (Tan et al., 2016b; Wong et al., 2016), yet all snakebite therapeutics (antivenoms) are manufactured from IgG of venom-immunized animals. The antineurotoxic efficacy of current antivenoms is further compromised because only 10–15% of the resulting IgG binds venom proteins (Casewell et al., 2010), with the remainder generated in response to environmental antigens to which the animals are exposed. Furthermore, antivenom efficacy is highly snake species-specific, resulting in limited cross-reactivity to venom toxins not included in the immunizing mixture (Tan et al., 2016a; Oh et al., 2019)—an inevitable result of venom variation (Williams et al., 2011). To circumvent this challenge, many antivenom manufacturers use mixtures of venoms as immunogens. However, this often results in polyspecific antivenoms requiring much higher therapeutic doses [e.g., 5–10 vials (50–100 ml) of ∼50–100 mg/ml antibodies] to effect cure, which, in turn, results in high incidences of foreign protein-related adverse reactions (de Silva et al., 2016) and prohibitively expensive treatment costs for impoverished snakebite victims [e.g., 48–315 USD/vial in Africa (Harrison et al., 2017)]. Therefore, a therapeutic approach providing cross-generic neutralization of snake venom neurotoxins (i.e., irrespective of the snake species responsible for the bite) and, at low therapeutic doses, would be highly valuable.

Acetylcholine binding proteins (AChBPs) are mimics of the extracellular ligand-binding domain of nAChRs and have been used extensively in structural studies of complexes of the receptor with a wide range of nicotinic agonists (Brejc et al., 2001), partial agonists (Hibbs et al., 2009), antagonists (Brams et al., 2011), and allosteric modulators (Spurny et al., 2015). AChBPs form pentameric complexes in which five subunits radially assemble around a central vestibule. The pharmacological properties of AChBPs from the freshwater snail *Lymnaea stagnalis* (Ls-AChBP) most closely resemble the human α7-nAChR (Smit et al., 2001), which is one of the most abundant nAChR subtypes in the brain. Other receptor mimics with different binding specificities have also been described, including a humanized version of the neuronal α7 receptor (α7-AChBP) (Li et al., 2011). In addition to AChBPs, the *Torpedo marmorata* (marbled electric ray) electric organ nAChR (Unwin, 2005) has been extensively studied as a model for the heteropentameric muscle-type nAChR (Miyazawa et al., 2003).

In parallel with structural studies on these nAChR homologues, short- and long-chain 3FTxs from snake venom, such as α-bungarotoxin from *Bungarus multicinctus* (many-banded krait) (Huang et al., 2013), α-cobratoxin from *Naja kaouthia* (monocled cobra) (Bourne et al., 2005), and erabutoxin from *Laticauda semifasciata* (black-banded sea krait) (Servent et al., 1997), have been employed as ligands to characterize the binding interfaces between toxins and nAChRs. In mutagenesis studies using recombinant or purified toxins in combination with the neuronal or the *Torpedo* nAChRs, short-chain 3FTxs were shown to interact with high affinity with the muscle-type receptor (Antil et al., 1999; Servent et al., 2000), while long-chain 3FTxs effectively bound to both the muscle and neuronal α7-nAChRs (Servent et al., 1997; Antil-Delbeke et al., 2000). Nevertheless, these studies employed a limited number of isolated snake toxins and thus did not expose these receptors to all of the various 3FTx isoforms present in a single venom to investigate their interactions. More recently, AChBPs have been utilized for profiling venom neurotoxicity using a microfluidic system, resulting in the detection of low-affinity binders, such as venom phospholipases A_2_ (PLA_2_), that interact with AChBP, in addition to 3FTxs (Slagboom et al., 2018). Other studies have reported the ability of a *N. kaouthia* PLA_2_ to block acetylcholine currents in *Lymnaea stagnalis* neurons or to compete with α-bungarotoxin for binding to *Torpedo* or α7-type receptors (Vulfius et al., 2014). The purified *Torpedo* nAChR has also been employed in *in vitro* assays to assess antivenom efficacy against the venoms of *N. kaouthia* (Thai monocled cobra), *N. naja* (Indian cobra), and *B. candidus* (Malayan krait) (Ratanabanangkoon et al., 2017, Ratanabanangkoon et al., 2018).

In this study, we aimed to assess the nAChR-binding specificities of toxins found in a variety of elapid snake venoms sourced from distinct geographic regions by employing two recombinant neuronal nAChR mimics: the Ls-AChBP and the humanized α7-AChBP. Using a ligand-fishing approach, we characterized the AChBP-bound and AChBP-unbound fractions from seven elapid venoms by SDS-PAGE and mass spectrometry. We find that AChBPs effectively capture long-chain 3FTxs from a variety of snake venoms but have little interaction with short-chain 3FTxs. We also evaluated whether AChBP mimics could be used as therapeutics against envenoming using *Naja haje* (Egyptian cobra) venom in an *in vivo* murine model of venom lethality. Our data suggest that nAChR mimics show potential as cross-species adjunct therapeutics against the life-threatening pathologies caused by certain postsynaptic venom toxins, although future optimization to broaden their specificity against other classes of neurotoxins will likely be required to fully exploit their therapeutic potential.

## Materials and methods

### AChBP Expression and Purification

The AChBP from *Lymnaea stagnalis* (Ls-AChBP) and α7-AChBP were expressed and purified as previously described (Spurny et al., 2015) with slight modifications. In brief, a C-terminal His-tagged fusion of the protein was expressed in *Sf*9 insect cells (a clonal isolate of *Spodoptera frugiperda* Sf21 cells) using the Bac-to-Bac baculovirus expression system (Invitrogen). AChBPs were purified from the expression medium by adding Ni Sepharose 6 Fast Flow beads (GE Healthcare). The beads were collected on a column, and impurities were washed with buffer containing 20 mM Tris–Cl (pH 8.0), 300 mM NaCl, and 40 mM imidazole. AChBP was eluted with the same buffer containing 300 mM imidazole. Fractions containing AChBP were pooled, concentrated, and further purified using size-exclusion chromatography on a Superdex 200 10/300 GL column (GE Healthcare) and a running buffer containing 20 mM Tris–Cl (pH 8.0) and 150 mM NaCl. Fractions corresponding to the pentameric protein were pooled, concentrated to 6 mg/ml, snap-frozen in liquid nitrogen, and stored at −80°C until further use.

### Snake Venoms

Venoms were sourced from either wild-caught specimens maintained in or historical venom samples stored in the Herpetarium of the Liverpool School of Tropical Medicine. The venoms selected were from diverse geographical localities and were from: *Naja haje* (Egyptian cobra, Uganda), *Naja naja* (Indian cobra, captive bred origin), *Naja kaouthia* (monocled cobra, captive bred, likely of Thai ancestry), *Bungarus caeruleus* (common krait, historical venom of Indian origin), *Dendroaspis viridis* (West African green mamba, Togo), *Micrurus fulvius* (eastern coral snake, historical venom of USA origin), and *Oxyuranus scutellatus* (coastal taipan, Australia). Crude venoms were lyophilized and stored at 4°C to ensure long-term stability. Before use, venoms were resuspended to 10 mg/ml in PBS (pH 7.4) and then further diluted to 1 mg/ml stock solutions (with PBS) for the described experiments.

### Transcriptomics and Molecular Data

We constructed venom gland transcriptomes from single individuals for *N. haje* (*n* = 1, Uganda, wild-caught), *N. naja* (*n* = 1, captive bred of Indian origin), and *N. kaouthia* (*n* = 1, captive bred of Thai origin), from glands dissected 3 days post-venom extraction, using methods previously described by our group (Pla et al., 2017; Ainsworth et al., 2018a; Whiteley et al., 2018). The venom of the animals used for transcriptomics was either used for direct comparison for the *N. kaouthia* specimen or contributed to the venom pools used in this study for *N. haje* (the venom of six snakes from Uganda) or *N. naja* (historical venom pool of an unknown number of donor snakes of Indian ancestry). Briefly, the snakes were euthanized using an overdose of pentabarbitone, and the venom glands were excised and immediately flash frozen in liquid nitrogen. The venom gland tissue was homogenized under liquid nitrogen using a pestle and mortar and a TissueRuptor (QIAGEN), and total RNA was extracted using the TRIzol Plus RNA Purification System (Thermo Fisher) protocol. Resulting RNA samples were DNAse treated (On-Column PureLink DNase; Life Technologies) and total RNA eluted in 30 µl nuclease-free water (Qiagen). The RNA then underwent two rounds of poly(A) selection using the Dynabeads mRNA DIRECT purification kit (Life Technologies) and was eluted in nuclease-free water before the quality and quantity of isolated RNA was assessed using a Bioanalyzer (Agilent). Subsequently, the sequencing library was prepared using 50 ng of enriched RNA using the Script-Seq v2 RNA-Seq Library Preparation Kit (epicenter), following 12 cycles of amplification. The sequencing library was then purified using AMPure XP beads (Agencourt), quantified using the Qubit dsDNA HS Assay kit (Life Technologies), and the size distribution assessed using a Bioanalyzer (Agilent). Sequencing libraries were multiplexed with others not reported in this study, and sequencing was performed on an Illumina MiSeq platform using 250 bp paired-end read technology (Centre for Genomics Research, University of Liverpool). Each of the three samples represented one-sixth of a sequencing lane. The ensuing read data were quality processed as previously described (Ainsworth et al., 2018a), resulting in the removal of adapter sequences (Cutadapt; https://code.google.com/p/cutadapt/) and low-quality bases (Sickle; https://github.com/najoshi/sickle). Paired-end read data were then assembled into contigs using VTBuilder (Archer et al., 2014) with the following parameters: minimum transcript length, 150 bp; minimum read length, 150 bp; and minimum isoform similarity, 96%. Assembled contigs were then annotated using BLAST2GO Pro v3 (Conesa et al., 2005) using the blastx-fast algorithm with a significance threshold of 1e−5 against NCBI’s nonredundant (NR) protein database (41 volumes, Nov 2015). Following annotation, contigs were assigned to one of the following three categories: toxins (contigs with blast annotations to sequences previously described as pathogenic toxins), nontoxins (contigs matching other sequences, such as housekeeping genes), and unidentified (those with no matches assigned or hits <1e−5). Toxin contigs were then manually analyzed using sequence alignments generated by the MUSCLE algorithm (Edgar, 2004) embedded within MEGA v7 (Kumar et al., 2016) for quality control purposes, including the merging of contigs that exhibited 100% identity to one another in overlapping regions >50 bp long (indicative of underclustering during assembly), the identification of open reading frames, and the removal of contigs containing stop codons within the coding region. Finally, the toxin encoding contigs were translated using MEGA v7 and ExPASy Translate (https://web.expasy.org/translate/) to generate species-specific toxin databases for proteomic analyses (see below). Thus, for the cobras and *D. viridis*, we used translations of venom gland transcriptomic data generated in this study or previously published by our group (Ainsworth et al., 2018a). For *N. kaouthia*, we only used translations of the venom gland transcriptome generated in this study (and not other previously published studies), as venom from this same specimen was used in our AChBP pulldowns. For *M. fulvius* and *O. s. scutellatus* we used existing sequence resources available on Uniprot (accessed July 2018) (Herrera et al., 2012; Vergara et al., 2014). Lastly, for *B. caeruleus*, we extracted all proteomic data present in Uniprot (Oh et al., 2017) and supplemented it with translations of toxins from a venom gland transcriptome of its congener, *B. multicintus* (Jiang et al., 2011), to increase coverage and breadth of 3FTxs. A supplementary fasta file containing all of the translated toxins encoding sequences, and all of the protein sequences sourced from Uniprot, that together were used for our proteomic identifications, is available as **Supplementary Data Sheet 2**. Raw sequencing reads and resulting *de novo* assemblies generated for the *N. haje*, *N. naja*, and *N. kaouthia* venom gland transcriptomes have been deposited in the NCBI Sequence Read Archive (SRA) and Transcriptome Shotgun Assembly (TSA) databases, respectively, and are linked to the BioProject identifier PRJNA506018.

### Capture and Purification of Venom Toxins Using Magnetic Beads

Ligand-fishing using AChBP was adapted from a previously published method (Pochet et al., 2011). Briefly, 20 µg of venom and 20 µg of recombinant α7-AChBP were brought up in binding buffer (1 mM KH_2_PO_4_, 3 mM Na_2_HPO_4_, 0.16 mM NaCl, 20 mM Tris–Cl, pH 7.5) to a final volume of 200 µl and incubated for 2 h at room temperature with gentle agitation. Another sample containing 10 µg of venom and 4.8 µg of α7-AChBP was also incubated alongside these samples but did not undergo separation using magnetic beads. Dynabeads (His-Tag isolation and Pulldown, Thermo Fisher Scientific, cat. No.: 10103D) were washed three times in binding buffer and resuspended at a final concentration of 20 mg/ml. Thirty microliters of Dynabeads were then added to each sample and incubated for another 15 min with gentle agitation. The beads were next separated using a magnetic stand, and the flow-through was recovered as the “unbound” fraction. The beads were washed four times with 200 µl wash buffer (50 mM sodium phosphate buffer pH 8.0, 300 mM NaCl, 0.01% Tween-20), allowing 2 min for magnetic separation. The bound AChBP and toxin-AChBP complexes were eluted in 12 µl of His elution buffer (300 mM imidazole, 50 mM sodium phosphate buffer pH 8.0, 300 mM NaCl, 0.01% Tween-20) and resuspended 1:1 in 2× SDS-PAGE gel loading dye under reducing or nonreducing conditions and loaded onto 15% SDS-PAGE gels. The unbound and control sample were concentrated up to ∼15 µl using a speedvac, after which they were resuspended 1:1 in 2× SDS-PAGE loading buffer. For the Ls-AChBP, the conditions were the same, with the exception that 50 µl of 40 mg/ml Dynabeads was used to capture the AChBP and the elution volume was 30 µl.

### One-Dimensional SDS-PAGE Gel Electrophoresis

We used SDS-PAGE gel electrophoresis to evaluate the bound and unbound fractions in each of our profiled venoms following the ligand-fishing assay. For each venom, we ran SDS-PAGE gels containing the following five lanes: 10 μg of venom, 2.5 μg of AChBP, a mixture containing 10 μg of AChBP + 4.8 μg venom, and the entire bound and unbound fractions. Samples were diluted 1:1 with a reducing or nonreducing loading buffer. Samples were then loaded onto 15% hand-cast SDS-PAGE gels alongside a protein marker (Broad Range Molecular Marker, Promega) and run at 120 V for 90–100 min using a Mini-PROTEAN Tetra System (Bio-Rad). The resulting gels were stained with Coomassie brilliant blue for 2 h and then destained (4.5:1:4.5 methanol/acetic acid/H_2_O) for visualization.

### Two-Dimensional SDS-PAGE Gel Electrophoresis

We used our previously described method (Ainsworth et al., 2018b; Calvete et al., 2018) to perform two-dimensional (2D) SDS-PAGE gel electrophoresis experiments using *N. haje* venom. Ligand fishing was scaled up appropriately using 100 μg of α7-AChBP and 100 μg of *N. haje* venom. The AChBP and venom were incubated for 30 min at room temperature, after which the purification was carried out as described above. The bound and unbound fractions were prepared for 2D gel electrophoresis using the ReadyPrep^™^ 2-D Cleanup Kit for isoelectric focusing (IEF) (Bio-Rad) as per the manufacturer’s instructions. Cleaned-up venom samples were then applied to 7 cm, pH 3–10, nonlinear IPG strips (Bio-Rad) using the ReadyPrep^™^ 2-D starter kit (Bio-Rad), as per manufacturer’s instructions and rehydrated overnight at room temperature. After rehydration, IEF was performed using a PROTEAN^®^ IEF Cell (Bio-Rad) with the manufacturer’s standard electrophoresis protocol for 7 cm IPG strips (default cell temperature = 20°C; maximum current 50 µA/strip; voltage = 250 V with linear ramp for 20 min; 4,000 V with linear ramp for 2 h; 4,000 V with rapid ramp for 10,000 V-h). After IEF, IPG strips were equilibrated (as per the ReadyPrep™ 2-D starter kit) and loaded onto Mini-PROTEAN TGX AnyKd precast gels (Bio-Rad) and run at 200 V for 35 min. Gels were then rinsed in water and stained with G-250 Coomassie blue stain (Bio-Rad) for 2 h to visualize the proteins.

### In-Gel Tryptic Digestion of Proteins

All visible protein bands under ∼20 kDa were excised from the 1D nonreducing and 2D gels for both the bound and unbound fractions (**Supplementary Figure 1**) and prepared for mass spectrometry. Gel slices were cut into small pieces and washed twice in washing buffer 1 [100 mM ammonium bicarbonate, 50% acetonitrile (ACN)], followed by three ACN washes and centrifuged at top speed in a microfuge to pellet the gel particles. The samples were then incubated in 40 μl reduction buffer (25 mM ammonium bicarbonate, 0.5% β-mercaptoethanol) for 40 min at 56°C to reduce the disulfide bridges and alkylated with 55 mM iodoacetamide for 20 min in the dark. Next, the gel slices were alternatively washed with washing buffer and ACN until they turned white (no residual dye left), after which they were dried in a vacuum centrifuge. Samples were trypsinized overnight at 37°C with 0.6 µg of trypsin (Sigma, cat. no.: T6567) per sample, after which 1 μl of 5% formic acid (FA) was added to the tubes to quench the digestion. The samples were sequentially extracted in 0.1% FA, 0.1% FA and 50% ACN, and finally 100% ACN, and moved to autosampler vials with glass inserts.

### NanoLC-MS/MS of Tryptic Digests and Data Analysis

The NanoLC separation of the tryptic digests was performed using an UltiMate 3000 RSLCnano system (Thermo Fisher Scientific). The autosampler was run in full-loop injection mode. The autosampler was set to a 1-µl injection volume, and after injection, the samples were separated on an analytical capillary column (150 mm × 75 µm) with Aqua C18 particles, packed in-house (3 µm particle size and 200 Å pore diameter; Phenomenex). The mobile phases comprised eluent A (98% H_2_O, 2% ACN, 0.1% FA) and eluent B (98% ACN, 2% H_2_O, 0.1% FA). The gradient program used for the separation was as follows: 2 min isocratic separation at 5% B, linear increase to 80% B in 15 min, 3 min isocratic separation at 80% B, down to 5% B in 0.5 min, and equilibration for 9 min. The column was kept at 30°C in the column oven. Detection was performed by a variable wavelength detector set at 254 nm followed by a Bruker Maxis q-TOF mass spectrometer (Bruker). The mass spectrometer was operated in positive-ion mode and had an electrospray ionization (ESI) source. The ESI source parameters for the MS instrument consisted of the following: source temperature, 200°C; capillary voltage, 4.5 kV; and gas flow, 10 l/min. Spectra were obtained at 1 spectrum/s in the 50–3,000 *m*/*z* range. MS/MS spectra were obtained in data-dependent mode using 35-eV collision energy in the CID collision cell. Bruker Compass software was used for instrument control and data analysis.

Mascot (Matrix Science) searches against our custom databases (see above, **Supplementary Data Sheet 2**) were used for protein identification of the analyzed tryptic digests. The following search parameters were used: ESI-QUAD-TOF as the instrument type, semiTrypsin as the digestion enzyme allowing for one missed cleavage, carbamidomethyl on cysteine as a fixed modification, amidation (Protein C-term) and oxidation on methionine as variable modifications, ± 0.05 Da fragment mass tolerance, and ±0.2 Da peptide mass tolerance.

In addition to providing details on the resulting toxin coverage derived from the recovered peptides (see **Supplementary Tables 1** and **2**), we transformed the resulting data to provide an overview of peptide matching by extracting the unique peptides identified in our Mascot searches and then calculating a score for each identified toxin as the number of unique peptides per protein divided by the number of amino acids in that protein.

### Molecular Analysis of 3FTxs

The bound and unbound 3FTxs from the various snake species were aligned in MEGA v7 using MUSCLE and then manually curated and validated. The resulting alignments were then exported and annotated in Jalview (http://www.jalview.org) for figure production.

### Assessing the Efficacy of AChBP *in vivo*

Animal experiments were conducted using protocols approved by the Animal Welfare and Ethical Review Boards of the Liverpool School of Tropical Medicine and the University of Liverpool, and they were performed in specific pathogen-free conditions under licensed approval of the UK Home Office and in accordance with the Animal (Scientific Procedures) Act 1986 and institutional guidance on animal care. Experimental design was based upon refined WHO-recommended protocols (Harrison et al., 2017), and the investigators conducting the experiment were blinded to the treatment each group of mice would receive.

The median lethal dose (venom lethal dose 50 [LD_50_]) of *N. haje* venom (0.43 µg/g body weight) used in this experiment was previously determined (Harrison et al., 2017). Similarly, the median effective dose (effective dose 50 [ED_50_]) of SAIMR polyvalent against 5 × LD_50_ *N. haje* venom was previously determined to be 71 µl (Harrison et al., 2017). Owing to limited amounts of AChBP, we used 2.5 × LD_50_ doses of *N. haje* venom (20.4 µg) in a modified version of the antivenom ED_50_ neutralization experiments (Ainsworth et al., 2018b). Groups of five male 18–22 g CD-1 mice (Charles River, UK) received experimental doses, which comprised either a) venom only (2.5 × LD_50_, 20.4 µg); b) venom and SAIMR polyvalent antivenom [either the “suboptimal” dose of 0.25 × ED_50_ (17.75 µl) or the “protective” dose of 0.75 × ED_50_ (53.25 µl)], both previously determined against 5 × LD_50_ venom doses; c) venom and “suboptimal” dose of antivenom and α7-AChBP (102 µg); d) “suboptimal” dose of antivenom only; or e) α7-AChBP (102 µg) only. All experimental doses were prepared to a volume of 200 µl in PBS and incubated at 37°C for 30 min before their intravenous injection *via* the tail vein. The mice were monitored for 6 h and euthanized upon observation of humane endpoints (seizure, pulmonary distress, paralysis). Deaths, time of death, and survivors were recorded, where “deaths/time of death” actually represents the implementation of euthanasia based on the defined humane endpoints.

For statistical analysis, we performed a one-way ANOVA with Tukey’s *post hoc* test on the means of the five independent measurements in GraphPad Prism7, using default parameters. It is worth noting that this model utilizes the intravenous delivery of venom in order to cause venom-induced lethality within an acceptable experimental time frame and to reduce unnecessary suffering in experimental animals. However, the intravenous delivery of venom does not accurately reflect a human snakebite, and thus, the therapeutic doses used in this study cannot be easily extrapolated to anticipate the therapeutic doses that might be required clinically.

## Results

### Ligand Fishing with Ls- and α7-AChBP Captures Low Molecular Weight Toxins

**Figure 1:**
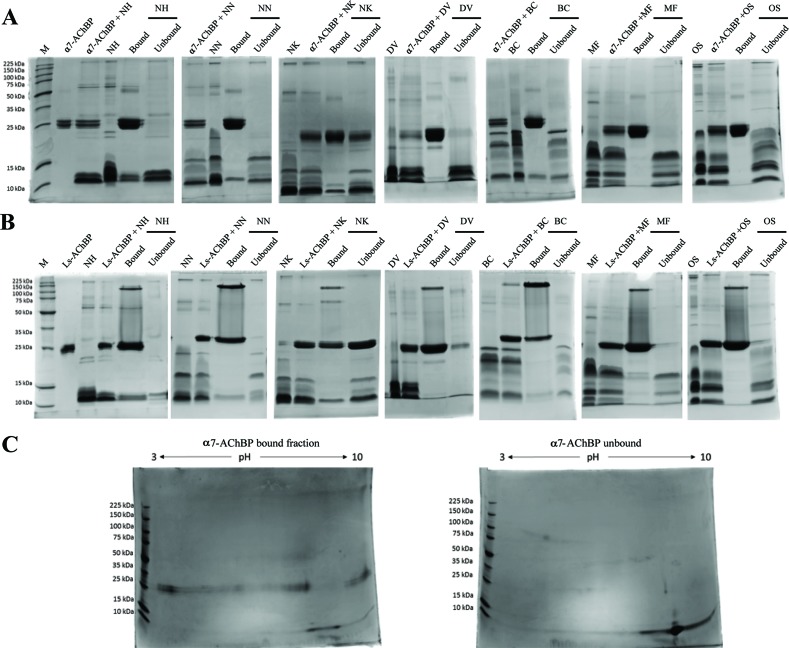
Toxins captured using the AChBP ligand-fishing assay. Nonreducing 1D and 2D SDS-PAGE gels showing the bound and unbound fractions from *N. haje* (NH), *N. naja* (NN), *N. kaouthia* (NK), *D. viridis* (DV), *B. caeruleus* (BC), *M. fulvius* (MF), and *O. s. scutellatus* (OS) venoms. The gels display the bound and unbound (FT) fractions from purifications using the α7-AChBP **(A)** and *Lymnaea stagnalis* AChBP (Ls-AChBP) **(B)** binding proteins. Venom alone and a mixture of both the venom and binding protein are also presented as controls. **(C)** 2D SDS-PAGE gels for the bound (left) and unbound fractions (right) collected from *N. haje* venom incubated with α7-AChBP.

To characterize the binding specificity of toxins from various neurotoxic elapid venoms to nAChRs, we used a ligand-fishing approach adapted from a previously published method (Pochet et al., 2011). Elapid venoms (*N. haje*, *N. naja*, *N. kaouthia*, *D. viridis*, *B. caeruleus, M. fulvius*, and *O. s. scutellatus*) were incubated with recombinant His-tagged nAChR mimics (α7-AChBP or Ls-AChBP), after which the toxin-bound fraction was purified using magnetic beads. The venom, AChBP, and bead amounts, as well as the incubation times, were optimized with *N. haje* venom (**Supplementary Figure 2**), a representative venom rich in 3FTxs (Malih et al., 2014). The bound and unbound fractions were then separated on SDS-PAGE gels under reducing and nonreducing conditions (**Figures 1A, B** and **Supplementary Figure 3**). The monomeric α7- or Ls-AChBPs were observed at ∼25 kDa, and some AChBP dimers and pentamers were noted under nonreducing conditions, which dissociated in the presence of a reducing agent. Moreover, while proteins in the 3FTx molecular weight range (∼7–12 kDa) were recovered in both the bound and unbound fractions, the patterns of these bands differed, suggesting that specific toxins may be differentially captured by these AChBPs. In addition, these patterns were not markedly different between the humanized α7-AChBP and the *Lymnaea* Ls-AChBP, suggesting that these two types of nAChR mimics may be capturing similar toxins. To better resolve the captured fraction from the flow-through (i.e., to test for the potential of multiple captured toxins being present in single protein bands), we chose the humanized α7-AChBP and *N. haje* venom and performed 2D gel electrophoresis. However, this approach did not additionally distinguish between the protein bands in the 3FTx molecular weight range (∼7–12 kDa), suggesting that our 1D-gel approach was appropriate for identifying toxins that bind to AChBPs (**Figure 1C**).

Notably, some AChBP leakage into the unbound fraction was observed with *N. kaouthia* venom (**Figure 1**). This may be due to components in the venom denaturing the binding protein or masking the His-tag, thus hindering the binding of the AChBP to the beads, as this phenomenon was apparent when using both α7-AChBP and Ls-AChBP for ligand fishing. To a lesser extent, this was also observed with venom from *D. viridis* (Ls fractionation). Importantly though, the leakage of these AChBPs into the supernatant does not appear to markedly affect the patterns of the captured toxins. Overall, our results suggest that the α7- and Ls-AChBPs differentially bind proteins in the 3FTx molecular weight range.

### Long-Chain 3FTxs Bind With High Affinity to AChBPs

To elucidate the differences between the bound and unbound toxin fractions, the relevant protein bands were isolated from SDS-PAGE gels and subjected to tryptic digestion and mass spectrometry. To identify the resulting peptides, we initially performed a Mascot search using the SwissProt database. However, as snake venom proteins are not extensively represented in this database, the quality of the recovered matches was suboptimal, with very few matches obtained for our venoms of interest, and typically assigned to toxins identified from related snake species. To resolve this identification bottleneck, we utilized translations of transcriptomic data sourced from the venom glands of our species of interest as custom databases for MS/MS Mascot searches (see **Supplementary Data Sheet 2**). To this end, we generated and analyzed venom gland transcriptomes and their translations for *N. haje*, *N. naja*, and *N. kaouthia*, whose venoms were either utilized directly in this study (*N. kaouthia*) or contributed to the venom pool employed in the AChBP experiments (*N. haje* and *N. naja*). Existing, transcriptomic [*D. viridis* (Ainsworth et al., 2018a)] or proteomic resources [*M. fulvius* (Vergara et al., 2014), *B. caeruleus* (Oh et al., 2017), and *O. s. scutellatus* (Herrera et al., 2012)] were used for the remaining species under investigation.

**Figure 2:**
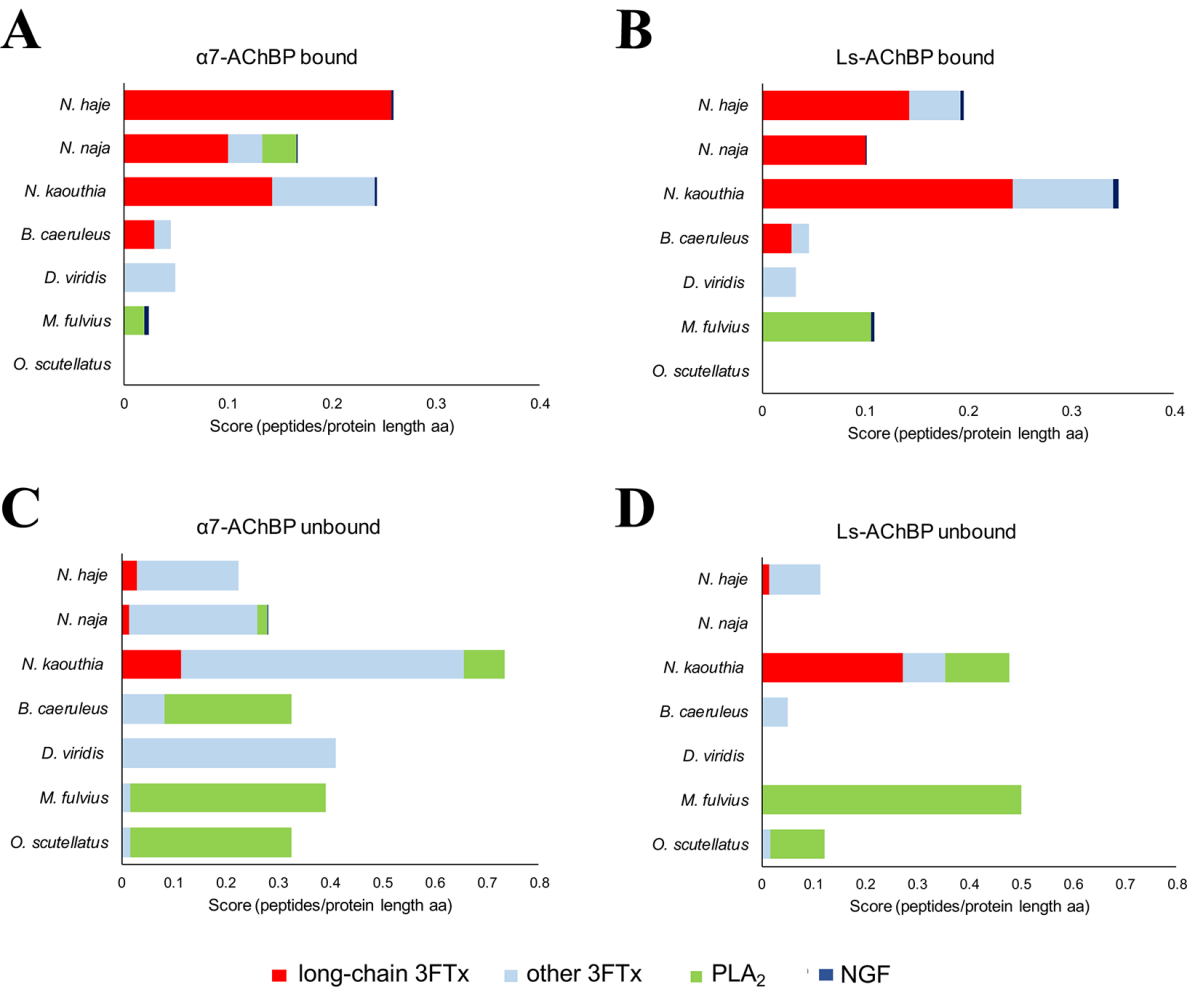
Identification of the toxins captured by α7- and Ls-AChBP. The mass spectrometry data were expressed as the number of unique peptides recovered per protein divided by the protein length (in amino acids) for all proteins in each of the bound and unbound fractions. Scores (number of peptides per protein length in aa) are displayed for the different classes of toxins captured from *N. haje*, *N. naja*, *N. kaouthia*, *D. viridis*, *B. caeruleus*, *M. fulvius*, and *O. s. scutellatus* venoms or recovered in the unbound fraction. Classes of toxins bound by α7-AChBP **(A)** or Ls-AChBP **(B)** are categorized into long-chain three-finger toxins (3FTxs), other 3FTxs, PLA_2_s, and NGF. Toxins recovered in the unbound fraction are presented in **(C)** (α7-AChBP) and **(D)** (Ls-AChBP). Please note that the axes have different lengths driven by the number of unique sequences recovered in each fraction.

In line with previous findings (Servent et al., 1997; Antil-Delbeke et al., 2000), our MS/MS identifications revealed that long-chain 3FTxs (long-chain α-neurotoxins) were consistently captured by both AChBPs when incubated with the three cobra venoms (*Naja* spp). and that of the common krait (*B. caeruleus*) (**Figure 2**, **Supplementary Tables 1**–**3**). Moreover, the protein IDs identified with both these AChBPs were largely consistent. In the cobra venoms, the detected peptides amounted to ∼60% sequence coverage for long-chain 3FTxs, while α-δ-bungarotoxin 4 was the top binder in *B. caeruleus* venom. In contrast, the highly similar short-chain 3FTxs (short-chain α-neurotoxins) were almost exclusively observed in the unbound fraction, or a similar number of peptides were identified in both the bound and unbound fractions (**Supplementary Table 3**), suggesting a rapid exchange between the on- and off-bead fractions. Thus, if these short-chain 3FTx isoforms are being captured, they are not tightly associated with AChBPs, suggesting either weak binding to the receptor or background noise. Bearing in mind the presence of AChBP in the flow-through when incubating with *N. kaouthia* venom, we observed a relatively equal distribution of long-chain 3FTxs between the bound and unbound fractions (e.g., 17 vs. 19 peptides for Ls-AChBP and 10 vs. 8 peptides for α7-AChBP recovered for long-chain 3FTx T1411, **Supplementary Table 3**), likely due to the association of some long-chain 3FTxs with an AChBP that cannot interact with the magnetic beads; however, this data nevertheless suggest that long-chain 3FTxs are still being captured in this venom.

In contrast to our results with venom from *Naja* spp. and *B. caeruleus*, no long-chain 3FTxs were recovered from the bound fractions following the incubation of the two AChBPs with *D. viridis* venom. We did detect one short-chain 3FTx bound to the AChBPs, but this toxin was also recovered in the unbound fraction (**Figure 2**). However, a previous analysis of the *D. viridis* venom gland transcriptome (Ainsworth et al., 2018a) demonstrated that while this species contains three long-chain 3FTx transcripts, all have very low expression (<0.1% of all venom toxins in the transcriptome). It is therefore unsurprising that these toxins were not detected *via* our approach. Furthermore, the short-chain 3FTx recovered from the bound fraction is the most highly expressed toxin in the *D. viridis* transcriptome (19.4% of all venom toxins) and represents 40.1% of the *D. viridis* proteome (Ainsworth et al., 2018a). The abundance of this short-chain 3FTx likely explains its transitory binding to AChBPs at high concentrations.

We did not observe any 3FTxs in the bound fractions from *M. fulvius* venom, and only one peptide from a short-chain 3FTx was recovered in the unbound fraction. Several PLA_2_ isoforms in this venom bound weakly to Ls-AChBP, but with one exception; we only recovered a few peptides for these toxins, while several PLA_2_ isoforms were observed in the flow-through. This low level of binding is potentially due to the high abundance of these toxins in the venom, as the *M. fulvius* venom gland transcriptome is dominated by PLA_2_s (∼65% of toxin expression), with only 21% of transcripts encoding 3FTxs (Margres et al., 2013). While the *M. fulvius* venom proteome was previously shown to contain 6–7 kDa nAChR-binding toxins, which made up 24.9% of the venom toxins, these toxins typically exhibited only weak and reversible binding to the muscle-type nAChR in a cell-based assay (Vergara et al., 2014). In the current study, we employed two types of α7-neuronal receptor mimics that appear to only be effectively binding long-chain, but not short-chain, 3FTxs across the species tested. Analysis of a sequence alignment of *M. fulvius* 3FTxs sourced from the venom gland transcriptome (Margres et al., 2013) revealed an absence of canonical long-chain 3FTxs (**Supplementary Figure 4**) and thus may explain these observations. Given that we only detect PLA_2_s from *M. fulvius* venom being weakly captured by the AChBPs, we conclude that these may be binding to Ls-AChBP at background levels, in a similar manner to previously reported interactions between viper or elapid PLA_2_s and this AChBP, which occur in the micromolar range (Vulfius et al., 2014; Slagboom et al., 2018).

Similarly, no 3FTx bands were recovered in the bound fraction of *O. s. scutellatus* venom, which is unsurprising as the *O. s. scutellatus* venom proteome consists of only 1.5% 3FTxs, while PLA_2_s represent 79.4% of all venom toxins (Herrera et al., 2012). In addition, the high neurotoxicity of this venom is driven by presynaptic neurotoxins (Kornhauser et al., 2010), among which taipoxin, a PLA_2_ heterotrimer, is the most toxic component isolated from snake venom to date. Upon visual inspection of all aligned 3FTxs in our dataset (**Supplementary Figure 5**), we did not identify any canonical long-chain 3FTxs. While long-chain 3FTxs (e.g., LNTX-1) have been proteomically annotated from *O. s. scutellatus* venom (Herrera et al., 2012), our analysis shows that this isoform does not contain the additional cysteines required to make the extra fifth disulfide bond characteristic of long-chain 3FTxs and, therefore, may have been misannotated based on its protein length. As with *M. fulvius*, our data imply that long-chain 3FTxs are not being captured from *O. s. scutellatus* because they may not be present in the venom.

Interestingly, we also identified venom nerve growth factor (vNGF) as an AChBP binder in several of the venoms surveyed. The toxic role of vNGF remains unclear, and it represents a minor venom component in almost all snake venoms from which it has been recovered (Tasoulis and Isbister, 2017). vNGF was effectively captured by both α7- and Ls-AChBP from the venoms of *N. haje*, *N. kaouthia*, and *M. fulvius*, while vNGF from *N. naja* venom only appeared to interact with Ls-AChBP. These results hint at the potential of vNGF to interact with nAChRs, although this requires future experimental validation.

### Bound 3FTxs Harbor Canonical Long-Chain 3FTx Features

**Figure 3:**
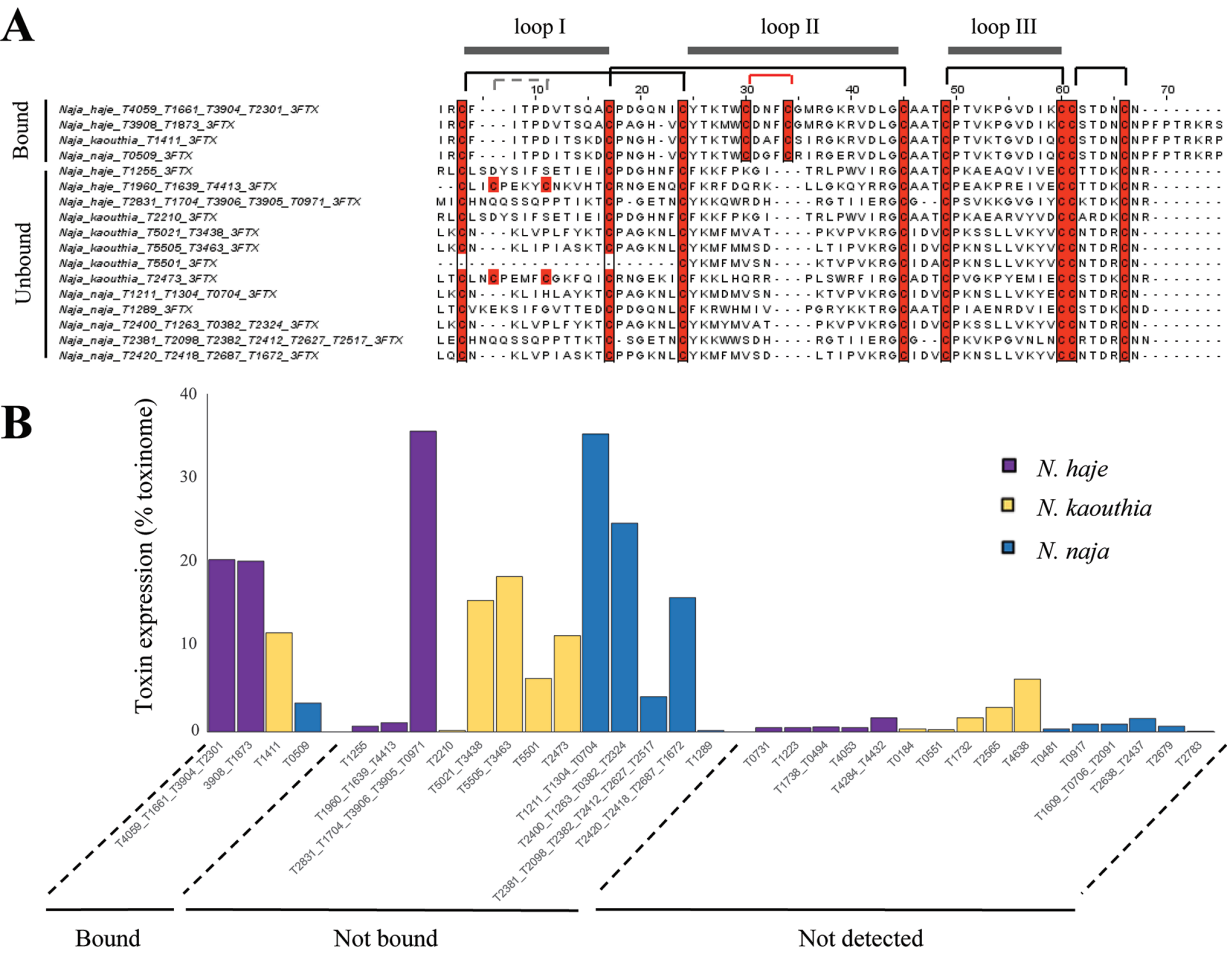
AChBPs capture available canonical long-chain 3FTxs. **(A)** Sequence alignment displaying 3FTxs captured (bound) and not captured (unbound) by AChBPs in the three *Naja* venoms. Cysteines are highlighted in red. The canonical disulfide bridges present in all 3FTxs are indicated by the black lines linking cysteines, while the gray lines highlight an additional disulfide bridge in loop I for two of the unbound toxins. The red lines indicate the additional cysteine residues that characterize long-chain 3FTxs as being distinct from other types of 3FTx. The three 3FTx loops are displayed above the alignment. **(B)** Comparisons of the transcriptomic expression of *Naja* toxins that were detected (both AChBP bound and unbound) or were not detected by mass spectrometry. Undetected toxins consistently had lower transcriptomic expression compared to those detected (whether in the bound or unbound fractions).

Our results suggest that AChBPs effectively capture long-chain 3FTxs and only weakly interact with other toxin classes, such as short-chain 3FTxs and PLA_2_s. To uncover the relevant features underlying the interaction of long-chain 3FTxs with AChBPs, we generated a sequence alignment of the 3FTxs recovered in the bound and unbound fractions of our ligand-fishing experiment, using the transcriptomic data (**Figure 3**). All bound toxins, with the exception of the *D. viridis* short-chain 3FTx described earlier, were confirmed as canonical long-chain 3FTxs, as they displayed high conservation throughout the second 3FTx loop and contained the two additional cysteine residues involved in the formation of the fifth disulfide bond (**Figure 3**). In contrast, all 3FTxs recovered in the unbound fraction were either short-chain 3FTxs, weak neurotoxins, or cardiotoxins.

### AChBPs as Therapeutics in a Murine Model of Envenoming

**Figure 4:**
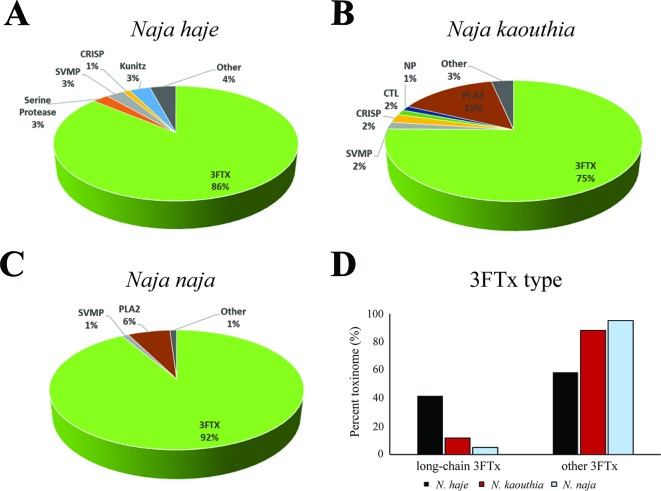
The relative expression of toxin families in the *Naja haje*, *N. naja*, and *N. kaouthia* venom gland transcriptomes. Breakdown of the expression levels of the toxins (toxinome) present in the *N. haje* **(A)**, *N. kaouthia* **(B)**, and *N. naja* **(C)** venom gland transcriptomes. **(D)** Comparisons of the relative expression of long-chain 3FTxs versus other 3FTxs in the venom gland transcriptomes (toxinome). 3FTx, three-finger toxins; SVMP, snake venom metalloproteinases; NP, natriuretic peptides; CTL, C-type lectins; PLA2, phospholipases A_2_; CRISP, cysteine-rich secretory proteins; Other, other minor expressed venom toxins.

**Figure 5:**
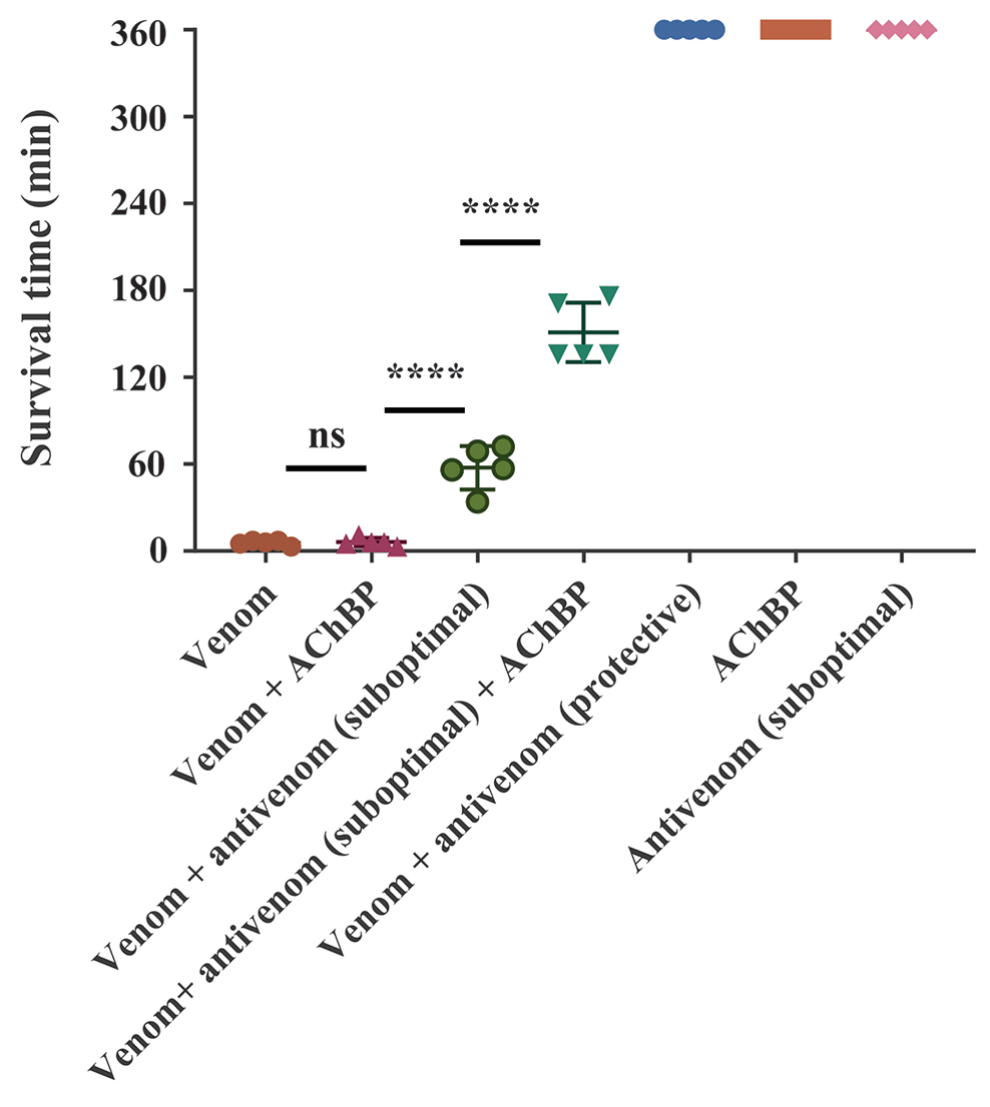
α7-AChBPs prolong survival *in vivo* when coadministered with antivenom. Groups of five mice were intravenously administered with either 2.5 × LD_50s_ of *N. haje* venom alone, AChBP alone, venom preincubated with AChBP (1:5 venom/AChBP ratio), a protective dose (53.25 µl) of SAIMR polyvalent antivenom alone, or venom preincubated with a protective dose of SAIMR polyvalent antivenom. A suboptimal dose (17.75 µl) of antivenom, which did not extend the survival of the experimental animals for more than 1 h, was also preincubated with 2.5 × LD_50s_ of venom and AChBP (1:5 venom/AChBP weight ratio) and delivered intravenously. Experimental animals were observed for the duration of the experiment (360 min) and survival times monitored. AChBP enhances the effect of antivenom when administered in combination with a suboptimal antivenom dose. Data points represent mean survival times and error bars represent SDs (*n* = 5). Ns, not significant, **** *P* < 0.0001, using a one-way ANOVA with Tukey’s test.

Our transcriptomic analysis (**Figure 4**) predicts *N. haje* venom to be highly enriched in long-chain 3FTxs, as the expression of these toxins constitutes ∼41% of that of all toxin-encoding genes expressed in the venom gland of this species. Since AChBPs effectively capture long-chain 3FTxs, we used *N. haje* venom as our model to test whether AChBPs could be used as a novel therapeutic approach for treating snake envenoming. To this end, we employed the gold-standard preclinical assay recommended by the World Health Organization (WHO, 2018) for assessing the efficacy of snakebite treatments (antivenoms) *in vivo*. We first challenged groups of mice (*n* = 5) with 2.5 × LD_50s_ of *N. haje* venom *via* the intravenous route and compared survival times with the same dose of venom preincubated with 102 µg of α7-AChBP (venom/AChBP = 1:5 ratio). We also administered the same dose of AChBP in the absence of venom, as a control. The absence of detectable adverse effects in the latter demonstrated that α7-AChBP is not acutely toxic to mice at this dose. However, we observed no difference in the mean survival times of the experimental animals that received venom alone and those that received venom preincubated with AChBP (6 vs. 6.2 min, respectively) (**Figure 5**). Given that AChBPs only captured long-chain 3FTxs in our ligand-fishing assays, these data suggest that either other toxic components present in the venom (e.g., short-chain 3FTxs, etc.) are also capable of causing lethality or that binding by AChBP does not result in the neutralization of long-chain 3FTxs.

To explore this further, we challenged mice with venom preincubated with an antivenom known to neutralize *N. haje* venom (SAIMR polyvalent antivenom) (Harrison et al., 2017). We incubated the same dose of venom with a “suboptimal” dose of antivenom (17.75 µl) unable to confer protection on its own and a “protective dose” predicted to prevent lethality (53.25 µl). In comparison with the venom-only control, the “protective” dose of antivenom provided complete protection against the lethal effects of the venom until the end of the experiment (360 min), while the “suboptimal” dose of antivenom prolonged survival by 51.6 min (**Figure 5**). The coincubation of *N. haje* venom with the “suboptimal” dose of antivenom and 102 µg of α7-AChBP prolonged the survival of the experimental animals for an additional 93.4 min, therefore resulting in increased survival for 145 min when compared to the venom-only control (**Figure 5**). These results demonstrate that the humanized α7-AChBP does confer some protection against venom toxins and thus is likely neutralizing at least some of the neurotoxicity caused by long-chain 3FTxs *in vivo*. It therefore seems likely that other toxic components found in the venom of *N. haje*, which do not interact with AChBPs, are responsible for causing the delayed lethality observed in this model.

## Discussion

Using a ligand-fishing approach to probe nAChR receptor mimics with a range of elapid snake venoms, we demonstrate herein that AChBPs interact with and capture long-chain 3FTxs but fail to bind to highly related 3FTxs, such as short-chain α-neurotoxins. Long-chain 3FTxs were recovered when using both the Ls-AChBP and the humanized α7-AChBP, which exhibit 24 and 64% identity with the human α7-receptor, respectively (Li et al., 2011); interestingly, these molecules specifically bound long-chain 3FTxs in a comparable manner. Our findings are consistent with prior studies that used purified long-chain 3FTxs and demonstrated that they interact with high affinity (*K*_d_ in the nanomolar range) with neuronal nAChR receptors, whereas short-chain 3FTxs only bind weakly (*K*_d_ in the micromolar range) (Servent et al., 1997).

Previous studies have highlighted the importance of the tip of the second 3FTx loop containing the fifth disulfide bridge (Servent et al., 1997;Bourne et al., 2005 ), and that of specific key residues in this loop (Antil-Delbeke et al., 2000), for the interaction between 3FTxs and the interface created between any two subunits of the Ls-AChBP. The reduction in this fifth disulfide bond in α-cobratoxin followed by dithiopyridylation lowered the affinity of this long-chain 3FTx for the α7-nAChR to a comparable level to that of short-chain neurotoxins (Servent et al., 1997), while a chimeric construct containing the fifth disulfide bond of long-chain neurotoxin 1 from *Naja oxiana* inserted into short-chain neurotoxin 2 from the same species was sufficient to increase the affinity of this toxin for the α7-nAChR to that of long-chain neurotoxins (Lyukmanova et al., 2007). Importantly, all 3FTxs that bound to the Ls- or α7-AChBP in the present study contained the fifth disulfide bond (**Figure 3**) and conserved Phe29 and Arg33 residues, which have previously been shown to interact with the Ls binding pocket (Bourne et al., 2005). In contrast, Phe29 was absent, and Arg33 was not conserved among the other 3FTxs recovered in the unbound fractions, nor did they contain the additional fifth disulfide bridge due to the absence of the two cysteine residues harbored by long-chain 3FTxs. Mutagenesis studies (Antil et al., 1999; Antil-Delbeke et al., 2000; Fruchart-Gaillard et al., 2002) have demonstrated that Trp25, Asp27, and Arg33 are also important residues for the binding of 3FTxs to both the neuronal and muscle-type receptors, while Ala28, Lys35, and the two cysteines involved in the fifth disulfide bond (Cys26 and Cys30) selectively bind to the α7-nAChR. Trp25, Asp27, and Arg33 were conserved across the captured long-chain 3FTxs identified here but not among the unbound 3FTxs recovered. In contrast, a neutral charge, but not the Ala28 residue itself, was conserved among the bound long-chain 3FTxs, while Lys35 was retained by all binders with the exception of the *N. naja* long-chain 3FTx. Moreover, α-δ-bungarotoxin 4 was the top AChBP binder from *B. caeruleus* venom, which is consistent with a recent report showing that α-δ-bungarotoxins interact with the α7-nAChR (Utkin et al., 2019).

Nonconventional 3FTxs can also bind to the neuronal α7-nAChR. One such example is candoxin (Nirthanan et al., 2002), a 66-amino-acid 3FTx neurotoxin from *B. candidus* that binds with high affinity to the α7-nAChR (IC_50_ = 50 nM) *via* a fifth disulfide bond present in loop I. This is in contrast to other weak neurotoxins from *N. kaouthia* (WTX) (Utkin et al., 2001) and *Naja sputatrix* (Wtn-5) (Poh et al., 2002) that bind only weakly (micromolar affinity) to this receptor, suggesting that other residues beside the fifth disulfide bond located in loop I dictate the binding affinity of these toxins to the neuronal receptor. Moreover, haditoxin (Roy et al., 2010), a homodimeric 3FTx from the venom of the king cobra, *Ophiophagus hannah*, was the first reported dimeric toxin to bind to α7-nAChR (IC_50_ = 180 nM). Given that haditoxin does not possess a fifth disulfide bond or residues important for contacting the α7-nAChR (e.g., Ala28, Lys35), it remains unclear how this dimer interacts with the neuronal receptor. These observations do suggest that while the fifth disulfide bond and specific residues that protrude into the binding pocket of the AChBP represent strong binding determinants, other contacts could drive the interaction of atypical 3FTxs with the α7-nAChR. However, in the present study, we did not detect the binding of any nonconventional toxins to AChBPs, likely because our ligand-fishing approach may only pull out the most abundant of binders. Toxin abundance clearly influences binding in our assay (**Figure 3**), as a highly abundant short-chain 3FTx from *D. viridis* venom was found to bind weakly to both the α7- and Ls-AChBPs, while PLA_2_s, which dominate the venom of *M. fulvius*, were also found to bind to the nAChR mimics.

Since nAChR mimics bind long-chain 3FTxs, which are some of the most potent neurotoxins found in elapid venoms [with LD_50_s ranging from 0.04 to 0.3 mg/kg (Nirthanan and Gwee, 2004)], we were interested in assessing the potential value of AChBPs as novel snakebite therapeutics for neurotoxic envenoming. We selected *N. haje* as our model for these studies, as this snake is considered to have a highly neurotoxic venom (Malih et al., 2014), and our transcriptomic analysis demonstrated that long-chain 3FTxs are the major venom components (∼41% of all toxins; **Figure 4**). While our *in vivo* data showed that the intravenous delivery of α7-AChBP was unable to protect mice from the lethal effects of *N. haje* venom, we demonstrated that these nAChR mimics are likely neutralizing long-chain 3FTxs, as increased survival times (*P* < 0.0001) were observed in envenomed mice treated with both α7-AChBP and a “suboptimal” dose of antivenom, when compared with the “suboptimal” dose of antivenom alone, which only briefly prolonged survival (**Figure 5**). Thus, the ability of α7-nAChR mimics to potentially neutralize a class of 3FTxs *in vivo* represents an encouraging prospect to build upon in the context of developing future treatments with the goal of generically targeting neurotoxicity in envenomed victims.

Given that the binding specificity of this molecule is directed predominately towards long-chain 3FTxs, broad-spectrum AChBPs capable of capturing a large variety of different 3FTx, or a mixture of AChBPs able to neutralize both short- and long-chain 3FTxs, would likely be needed to effect cure in envenomed victims. One advantage of the humanized α7-AChBP used in this study is its similarity (∼71%) with the human α7-nAChR, which may result in reduced immunogenic effects if administered to patients. The engineering of broad-spectrum AChBPs would ideally also use this humanized molecular backbone while taking advantage of the increased affinity (Nirthanan and Gwee, 2004) and pharmacological properties of other nAChR mimics, such as the *Torpedo* nAChR. Finally, while we explored the toxin capturing potential of AChBPs against a number of geographically diverse elapid snakes, such optimized broad-spectrum AChBPs would require testing against an increased breadth of venoms, including additional species within the elapid genera studied here and perhaps also members of the Hydrophiinae (marine elapids), whose venoms often contain abundant and highly potent α-neurotoxins that are typically not effectively neutralized by existing antivenoms (Tan et al., 2015; Tan et al., 2018).

Irrespective of whether a single broad-spectrum nAChR mimic or multiple mimics with different specificities would be required, such approaches could prove highly advantageous for circumventing venom variation—a major limitation with current antivenoms, which are typically only effective against a single, or a limited number of, snake venom/s. By neutralizing a whole class of toxins, a successful receptor mimic would thus represent a significant step forward compared to existing polyclonal antivenoms, which, given their low economies of scale due to restricted geographical usage, result in high costs to impoverished snakebite victims (Gutiérrez et al., 2017). Conceptually, this approach is analogous to recent studies exploring other non-antibody therapeutics, such as enzyme inhibitors and metal chelators (Ainsworth et al., 2018b; Bulfone et al., 2018), to target whole classes of snake venom toxins that cause pathologies such as hemorrhage and coagulopathy. Ideally, these AChBP mimics would bind rapidly and with high affinity to venom toxins, thus precluding their binding to nAChRs in the victim. Strong and rapid binding is a highly desirable characteristic for new therapeutics, as delays in treatment in resource-poor settings inevitably allow 3FTxs to access nAChR receptors before treatment administration. Therefore, investigating whether AChBPs can effectively displace already bound toxins should be a key focus of future research. Nonetheless, our present *in vivo* results show the promise of AChBPs to delay lethality caused by neurotoxic snake venom and thus advocate exploring such characteristics in future work.

As an alternative to the use of AChBPs as solo therapeutics, the coadministration of these molecules alongside traditional antivenom therapy may prove to be of greatest value. Prolonged survival was observed in our mouse model of envenoming when AChBP was administered alongside a suboptimal dose of polyvalent antivenom. These findings suggest that AChBPs might facilitate reducing clinical doses of antivenom in the future, as only 10–15% of antivenom immunoglobulins are typically specific to venom toxins and only a proportion of those antibodies will be specific to highly pathogenic toxins (e.g., 3FTx neurotoxins in this case) found in any particular venom (Gutiérrez et al., 2017). As many antivenoms require large therapeutic doses to effect cure, the further development of a highly specific yet generically acting (e.g., irrespective of snake species) inhibitor capable of reducing antivenom doses should result in a decreased incidence of adverse effects associated with antivenom administration and, most importantly, reduce treatment costs.

In an analogous manner, a recent study has demonstrated the value of the PLA_2_ inhibitor varespladib, alone or in combination with antivenom, in preventing the onset of neurotoxicity after the administration of the neurotoxic *O. s. scutellatus* venom in experimental animals (Lewin et al., 2018). While our model venom (*N. haje*) contains only trace amounts of PLA_2_s (< 0.05% of toxins in the venom gland transcriptome), this class of enzymes has been found to be more abundant in other cobra species (**Figure 4**) (Tan et al., 2019) and indeed other elapid snakes, such as *O. s. scutellatus* and *M. fulvius* (Herrera et al., 2012; Vergara et al., 2014). PLA_2_s may enable and accelerate the spread of the venom after a bite *via* their cytolytic effects (Gutiérrez and Ownby, 2003), thus enhancing the access of other venom components to their targets. In the case of elapid snakes, PLA_2_s have also been demonstrated to potentiate the cytotoxic effect of specific 3FTxs (Suzuki-Matsubara et al., 2016) and may facilitate the rapid action of 3FTxs on their muscular and neuronal targets, leading to the onset of paralysis. Therefore, combination treatments using AChBPs, enzyme inhibitors such as varespladib, and/or antivenom should be extensively explored by the scientific community to address current therapeutic limitations associated with this lethal neglected tropical disease.

In summary, our study demonstrates a proof of principle that AChBP-type proteins can effectively capture entire classes of lethal toxins, irrespective of variation in venom composition among snake species, and that they may possess therapeutic potential when administered in combination with antivenom. Further work is required to determine if broad-spectrum AChBPs can be engineered or whether combinations of different AChBPs offer a viable strategy to combat neurotoxicity in envenomed snakebite victims. Further studies are also required to define the likely effective dose of these optimized molecules and whether they are likely to be well tolerated by humans. Despite saving thousands of lives annually, the numerous limitations associated with existing snakebite treatments (e.g., species specificity, limited cross-reactivity, severe adverse reactions, high cost) strongly advocate for research into alternative or adjunct treatments that could be used to treat snakebite caused by a wide variety of medically important snake species. AChBPs are promising in this regard, and their subsequent development and validation may lead to them becoming a welcome addition to the snakebite treatment portfolio.

## Ethics Statement

All animal experiments were conducted using protocols approved by the Animal Welfare and Ethical Review Boards of the Liverpool School of Tropical Medicine and the University of Liverpool, and they were performed in specific pathogen free conditions under licensed approval of the UK Home Office and in accordance with the Animal [Scientific Procedures] Act 1986 and institutional guidance on animal care. Experimental design was based upon refined WHO-recommended protocols.

## Author Contributions

L-OA, CU, JK, and NRC devised the study. CU expressed and purified the AChBP proteins. L-OA performed the AChBP ligand-fishing experiments and prepared the samples for mass spectrometry. JS and BB performed mass spectrometry experiments. L-OA, JS, BB, JK, and NRC analyzed the resulting data. TK, GW, SCW, and NRC generated and analyzed the transcriptomes. SA, JA, RAH, and NRC performed the *in vivo* experiments. L-OA wrote the manuscript, with assistance from CU, JK, and NRC, and input from all other authors.

## Funding

This work was supported by a UK Medical Research Council grant (MR/L01839X/1) to RAH and NRC, a Leverhulme Trust research grant (RPG-2012-627) to RAH, a KU Leuven C3-project (C32/16/035) to CU and a Wellcome Trust and Royal Society Sir Henry Dale Fellowship (200517/Z/16/Z) to NRC.

## Conflict of Interest Statement

The authors declare that the research was conducted in the absence of any commercial or financial relationships that could be construed as a potential conflict of interest.

The handling editor is currently editing/co-organizing a Research Topic with one of the authors, CU, and confirms the absence of any other collaboration.
