# Supplementary material for: A Decoy-Receptor Approach Using Nicotinic Acetylcholine Receptor Mimics Reveals Their Potential as Novel Therapeutics Against Neurotoxic Snakebite
Source: Front Pharmacol. 2019 Jul 30;10:848. doi: 10.3389/fphar.2019.00848 (PMC6683245; doi:10.3389/fphar.2019.00848)
Supplement: Supplementary file 1 [file DataSheet_1.docx]

Supplementary Material

**Supplementary files**

**Supplementary File 1**: Fasta file containing all translated toxin sequences (*N. haje, N. naja, N. kaouthia, D. viridis, B. multicinctus*) and all proteomic data for the available species (*M. fulvius*, *B. caeruleus,* and *O. scutellatus*) used for proteomic identifications.

**Supplementary File 2**: Mass spectrometry data displaying the bound and unbound peptides following the incubation of venoms with α7-AChBP

**Supplementary File 3**: Mass spectrometry data displaying the bound and unbound peptides following the incubation of venoms with Ls-AChBP

**Supplementary File 4**: The number of unique peptides captured by α7- or Ls-AChBP, detailed by toxin class. The heatmap indicates low (yellow) to high (green) abundance. Long-chain 3FTxs are defined by the presence of the 5^th^ disulfide bond.

**Supplementary figures**


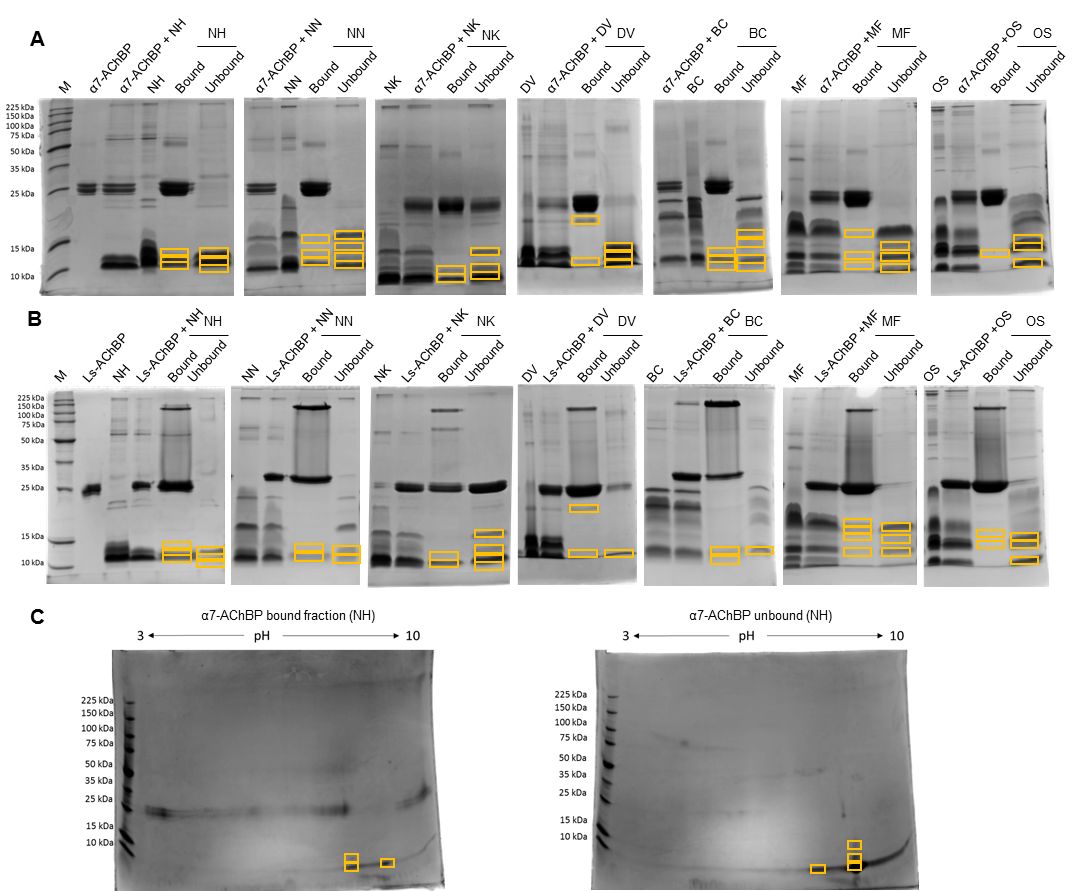


**Supplementary Figure 1. Bands excised from gels for mass spectrometry.**

Nonreducing 1D and 2D SDS-PAGE gels showing the bound and unbound fractions from *N. haje* (NH), *N. naja* (NN), *N. kaouthia* (NK), *D. viridis* (DV), *B. caeruleus* (BC), *M. fulvius* (MF) and *O. s.* *scutellatus* (OS) venoms. The gels display the bound and unbound fractions from purifications using the α7-AChBP (**A**) and Ls-AChBP (**B**) binding proteins. Venom alone and a mixture of both the venom and binding protein are also presented as controls. (**C**) 2D SDS-PAGE gels for the bound (left) and unbound fractions (right) collected from *N. haje* venom incubated with α7-AChBP. Protein bands excised are highlighted by yellow boxes.

**
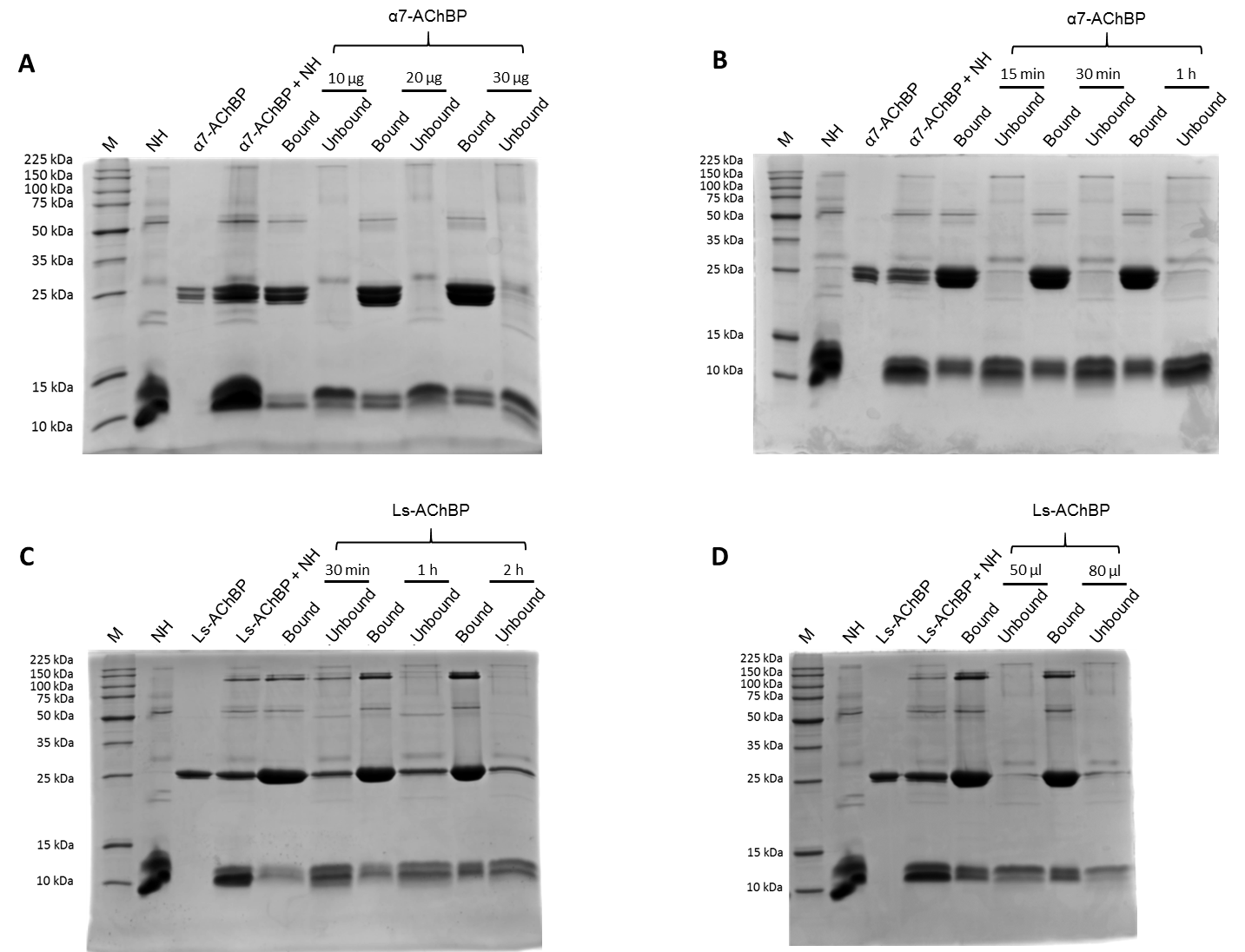
**

**Supplementary Figure 2. Optimization of venom and AChBP-toxin binding conditions**

The amounts of venom and AChBP were independently optimized for the α7- and Ls-AChBPs using *Naja haje* (NH) venom. Venom alone (10 µg), AChBP alone (4.8 µg), a mix of the two, and the entire bound and unbound fractions following our ligand-fishing assay (see Materials and Methods) were run on 15% SDS-PAGE gels under nonreducing conditions. Various amounts of α7-AChBP (10-30 µg) **(A)** were incubated with 20 µg of venom. A 1:1 ratio (20 µg venom:20 µg of binding protein) was chosen for further experiments. The binding time of the toxins to the AChBP was also optimized (15 min-1 h) **(B)**, which showed that 15 minutes appeared sufficient for the binding of α7-AChBP to venom toxins. However, for consistency, we conservatively set the time for the assay at 2 h, as the binding of Ls-AChBP to venoms toxins required an extended period of time (2 h) **(C)**. The binding of Ls-AChBP to venom toxins was modelled based on our α7-AChBP results. Thus, 20 µg of venom and a binding time of 2 h were used in the assay. However, as seen in panel (**C)**, Ls-AChBP was not entirely captured by the original quantity of Dynabeads used (30 µl of 20 mg/ml). Therefore, we tested increasing concentrations (40 mg/ml) and volumes of beads (**D**) and ultimately used 50 µl of 40 mg/ml beads to capture the entire protein pool.

**
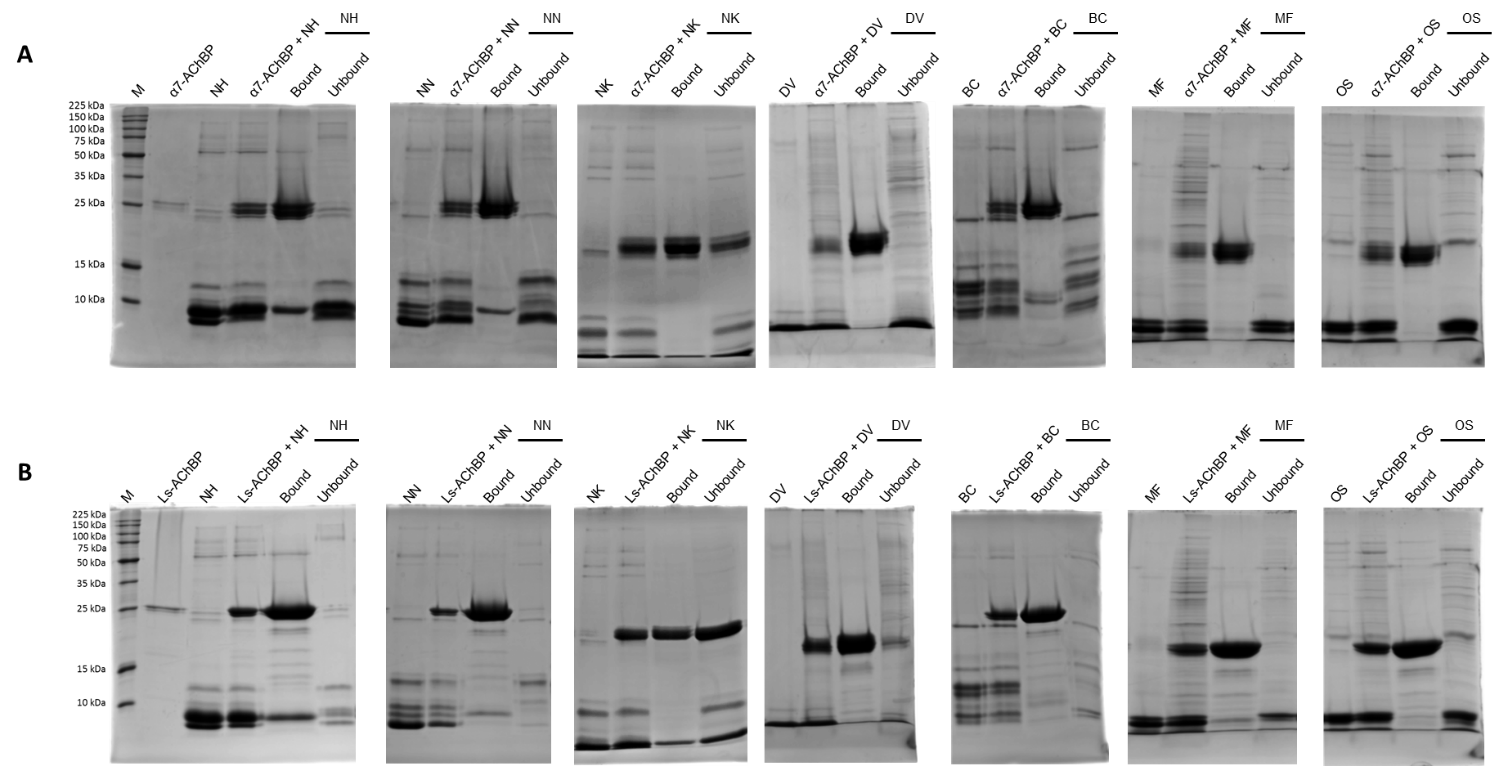
**

**Supplementary Figure 3. Toxins captured using the AChBP ligand-fishing assay (reducing conditions)**

SDS-PAGE gels run under reducing conditions showing the bound and unbound fractions from *Naja haje* (NH), *Naja naja* (NN), *Naja kaouthia* (NK), *Dendroaspis viridis* (DV), *Bungarus caeruleus* (BC), *Micrurus fulvius* (MF) and *Oxyuranus scutellatus* (OS) venoms. The gels display the bound and unbound fractions from purifications using the α7-AChBP **(A)** and Ls-AChBP **(B)**. Venom alone and a mixture of both the venom and binding protein are also presented as controls.

**
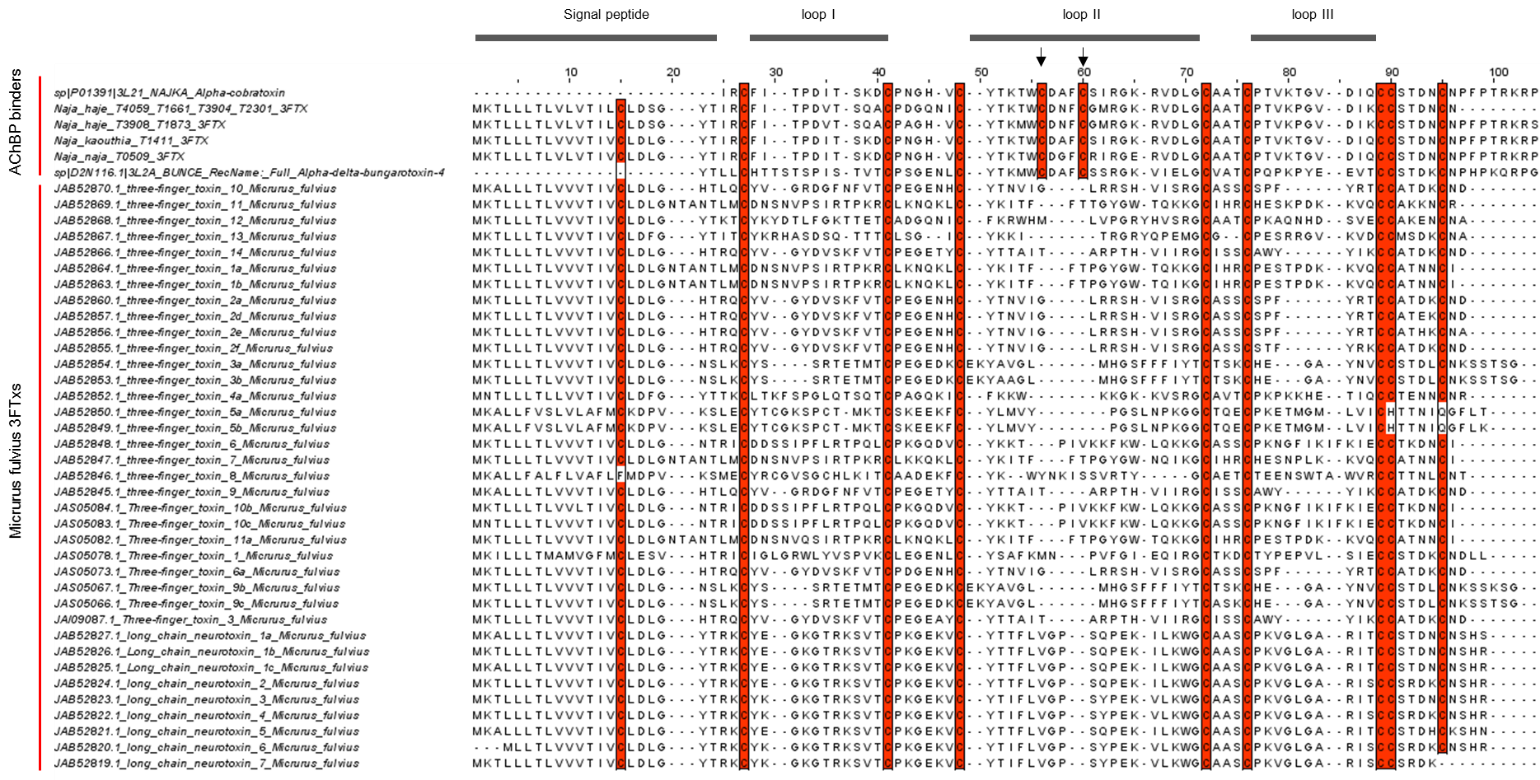
**

**Supplementary Figure 4. *Micrurus fulvius* three-finger toxin (3FTx) alignment**

Alignment of all *M. fulvius* 3FTxs with long-chain 3FTxs that we detected bound to AChBPs in this study (from other snake species). The additional cysteines present in the bound long-chain 3FTxs are indicated by arrows. As observed, there are no canonical long-chain 3FTxs present in the *M. fulvius* transcriptome, as even those annotated as long-chain 3FTxs lack the characteristic fifth disulfide bond in loop II.

**
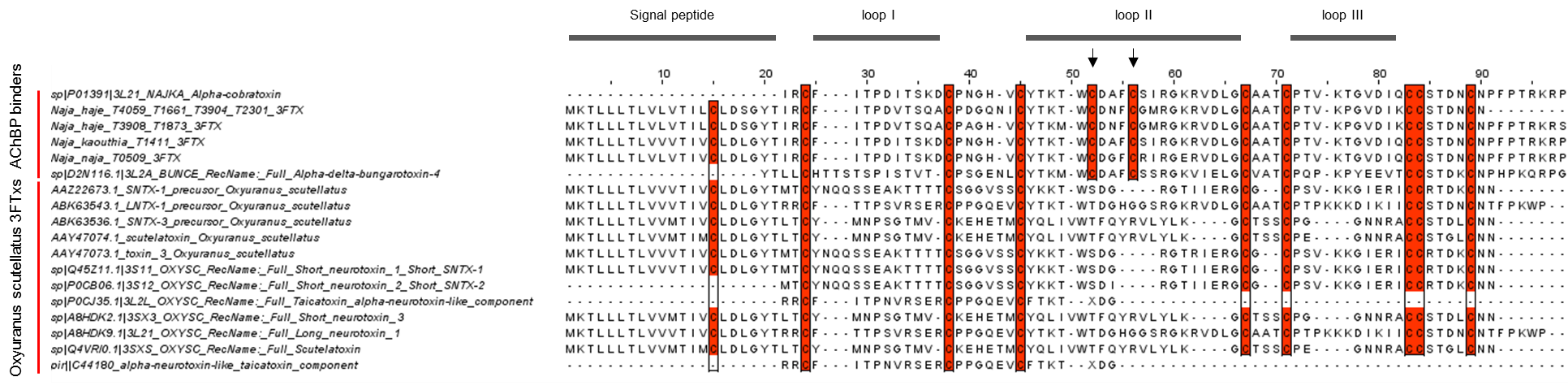
**

**Supplementary Figure 5. *Oxyuranus scutellatus scutellatus* three-finger toxin (3FTx) alignment**

Alignment of all *O. s. scutellatus* 3FTxs with long-chain 3FTxs that we detected bound to AChBPs in this study (from other snake species). The additional cysteines present in the bound long-chain 3FTxs are indicated by arrows. As observed, there are no canonical long-chain 3FTxs identified in the venom of *O. s. scutellatus*.
